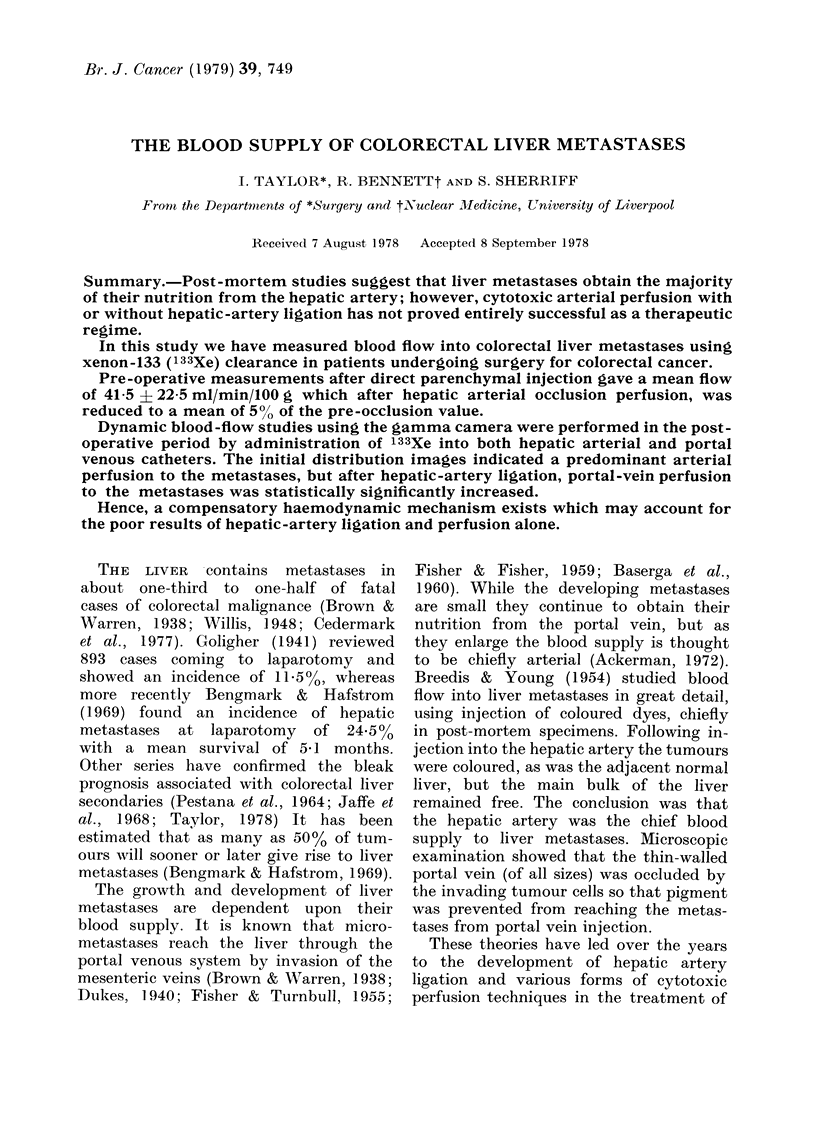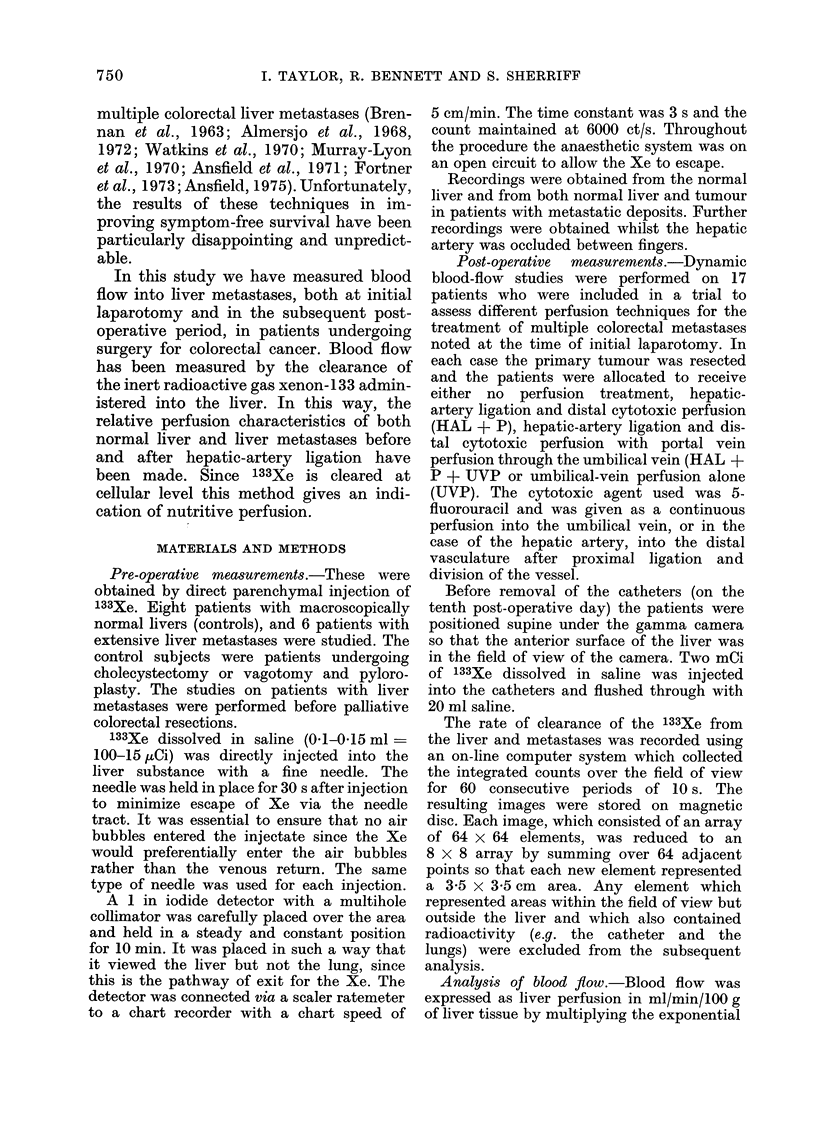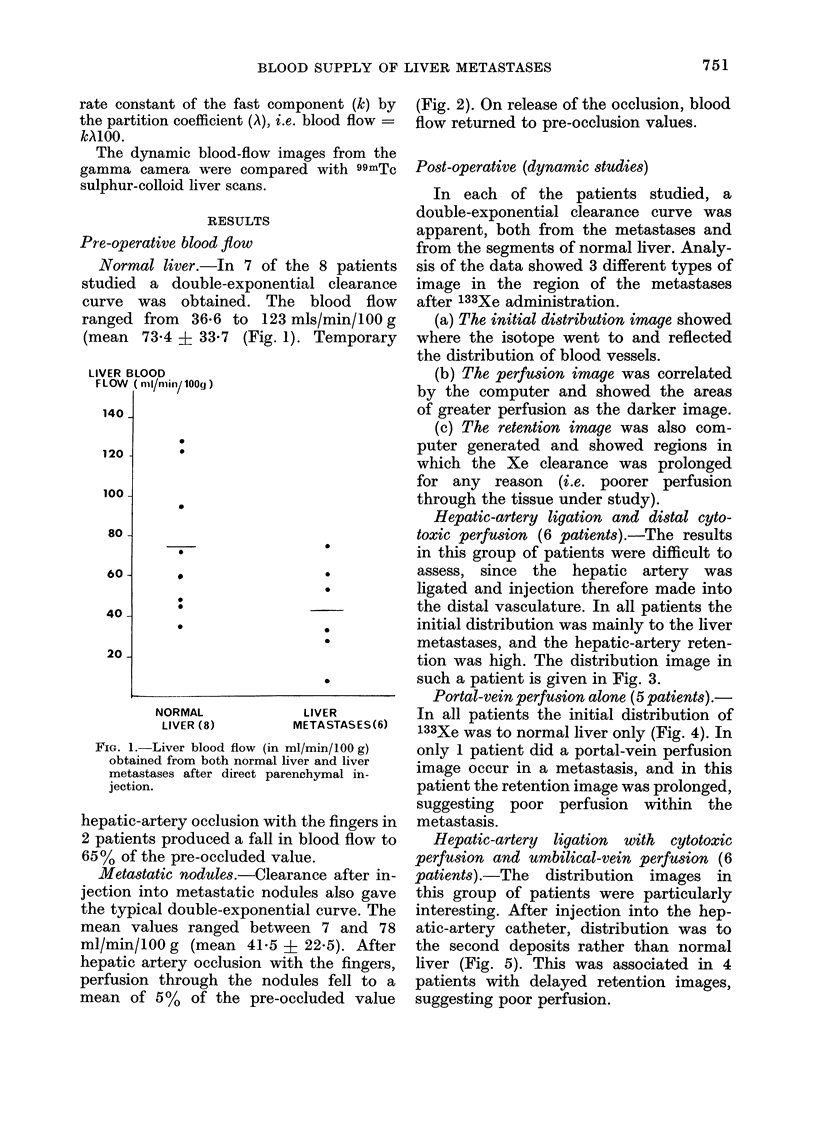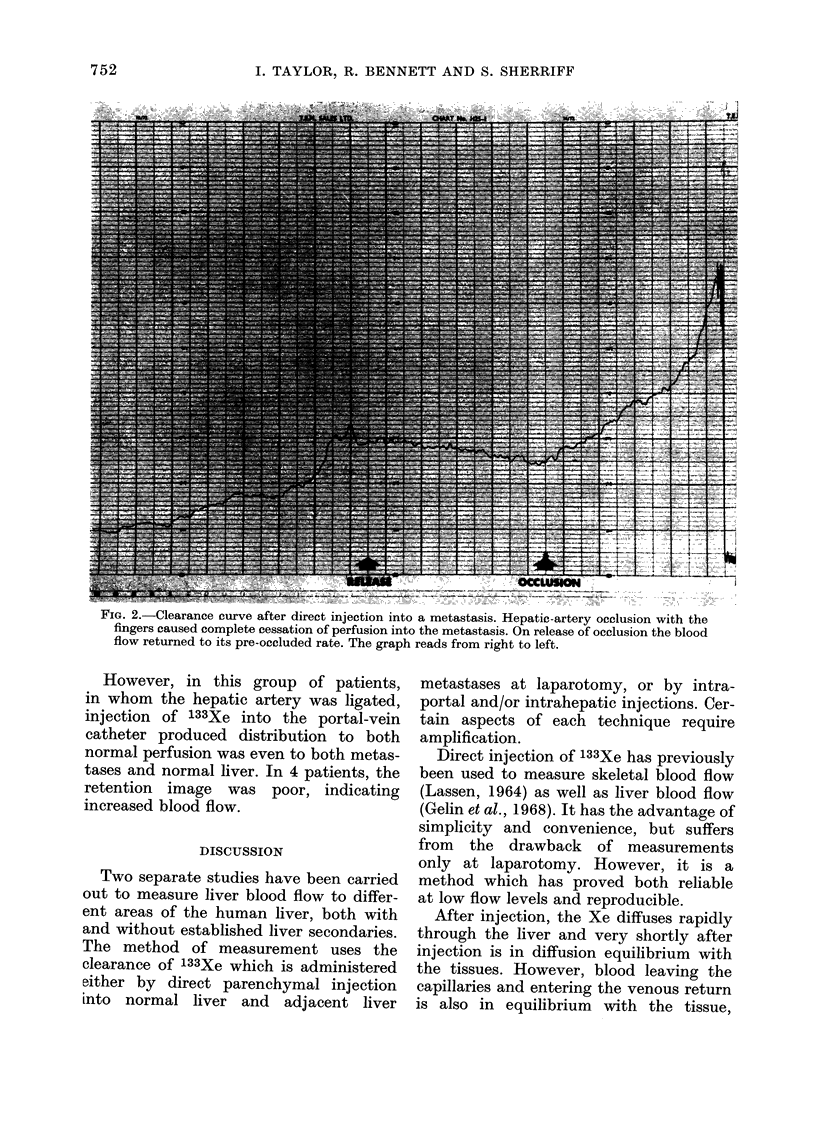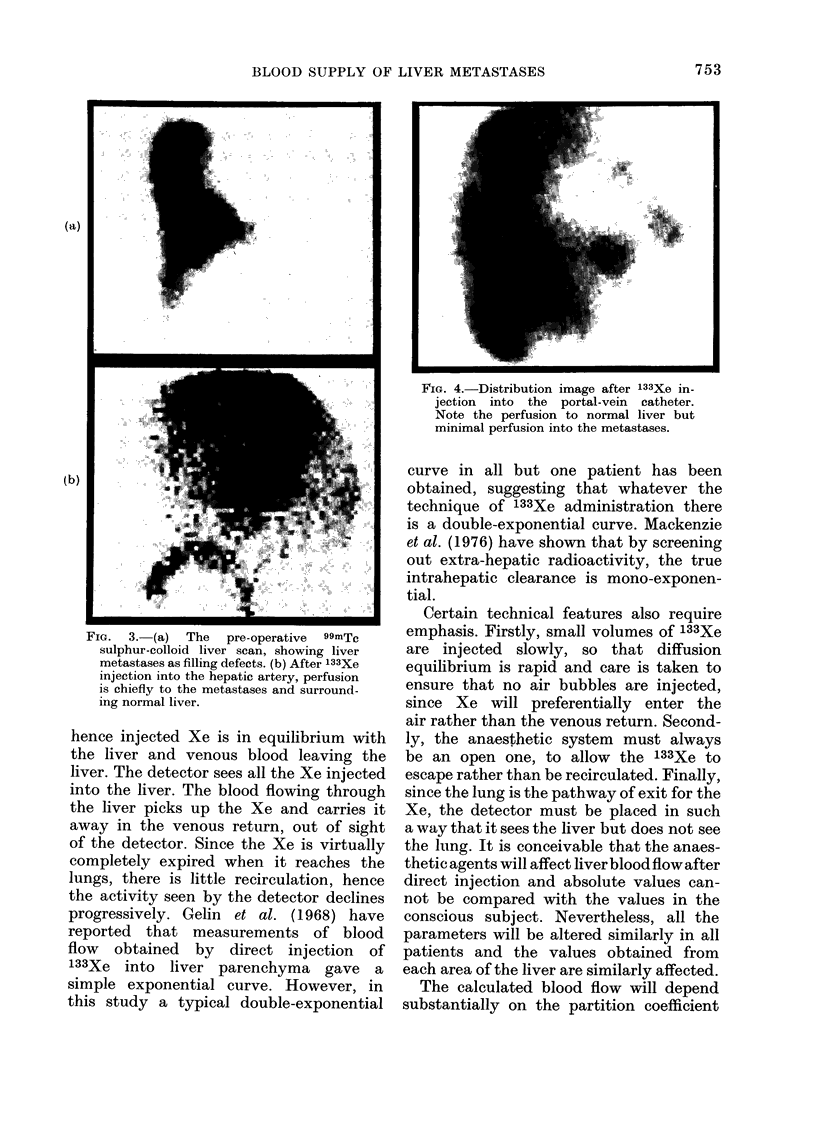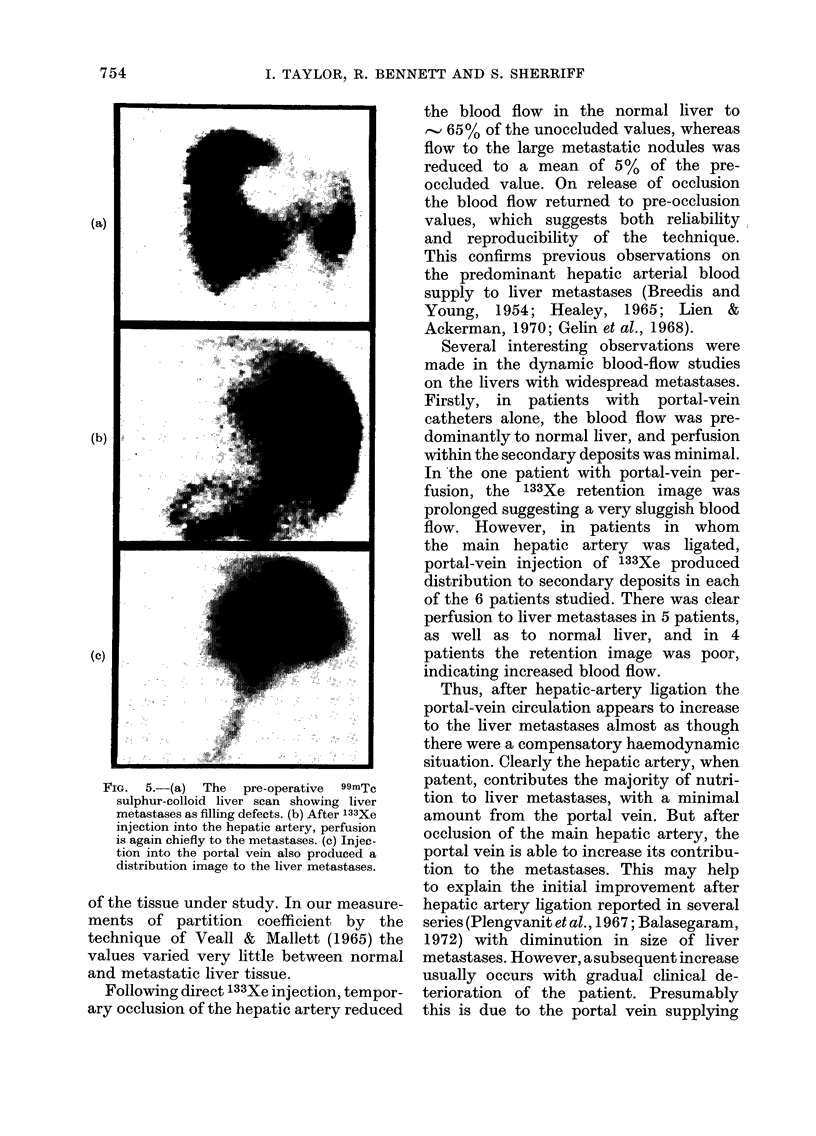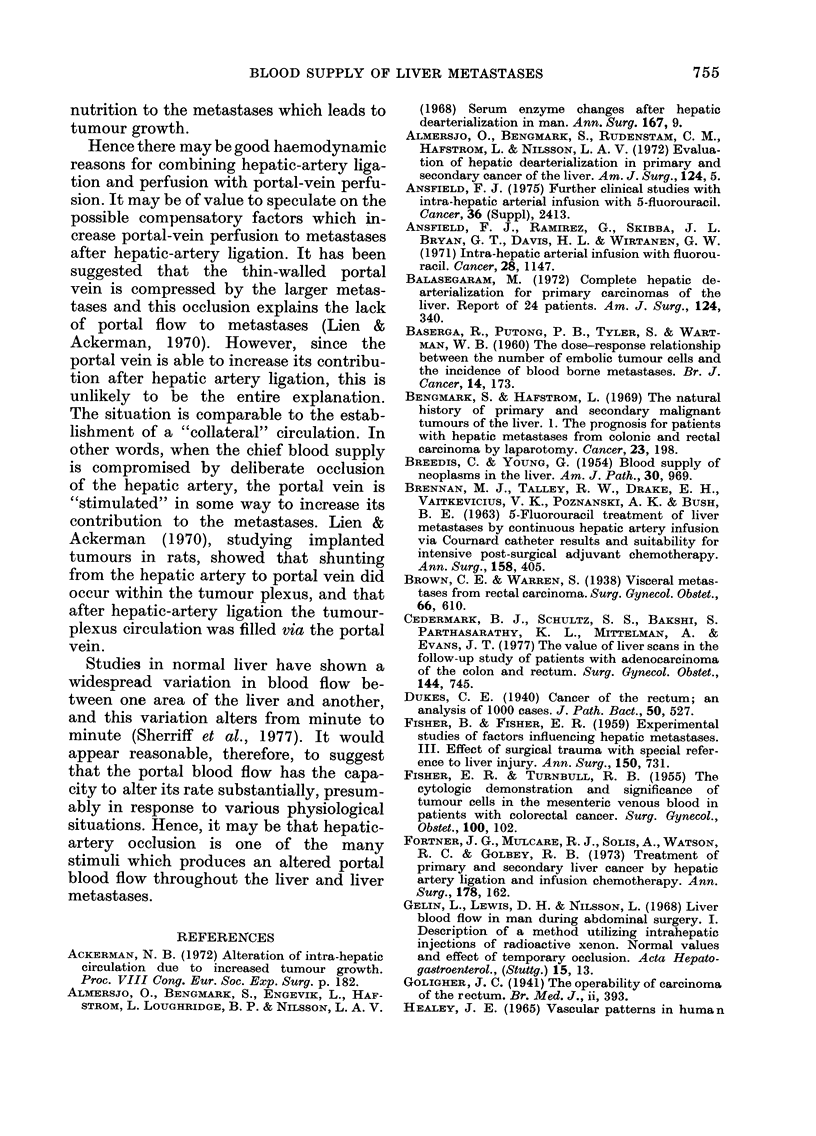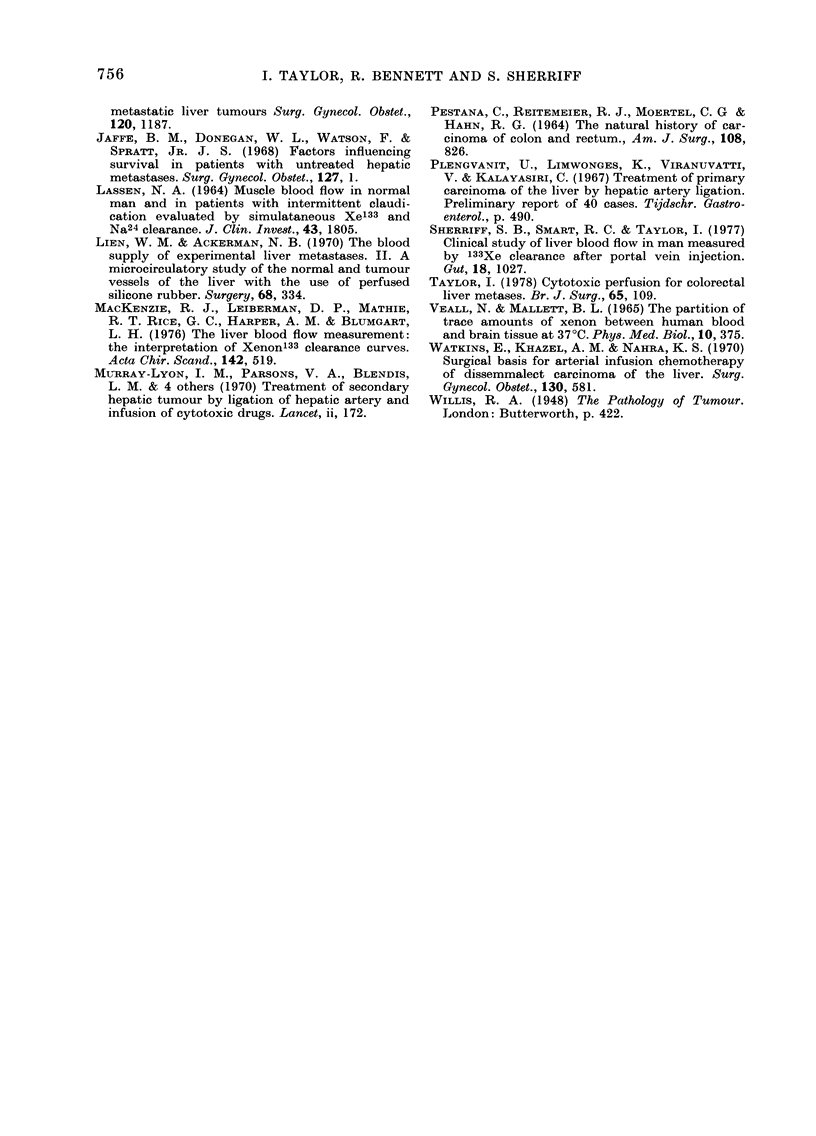# The blood supply of colorectal liver metastases.

**DOI:** 10.1038/bjc.1978.283

**Published:** 1978-12

**Authors:** I. Taylor, R. Bennett, S. Sherriff

## Abstract

**Images:**


*Br. J. Cancer* (1979) **39**, 749

### THE BLOOD SUPPLY OF COLORECTAL LIVER METASTASES

I. TAYLOR*, R. BENNETT† AND S. SHERRIFF

*From the Departments of *Surgery and †Nuclear Medicine, University of Liverpool*

Received 7 August 1978 Accepted 8 September 1978

**Summary.—Post-mortem studies suggest that liver metastases obtain the majority of their nutrition from the hepatic artery; however, cytotoxic arterial perfusion with or without hepatic-artery ligation has not proved entirely successful as a therapeutic regime.**

**In this study we have measured blood flow into colorectal liver metastases using xenon-133 ($^{133}$Xe) clearance in patients undergoing surgery for colorectal cancer.**

**Pre-operative measurements after direct parenchymal injection gave a mean flow of 41·5 ± 22·5 ml/min/100 g which after hepatic arterial occlusion perfusion, was reduced to a mean of 5% of the pre-occlusion value.**

**Dynamic blood-flow studies using the gamma camera were performed in the post-operative period by administration of $^{133}$Xe into both hepatic arterial and portal venous catheters. The initial distribution images indicated a predominant arterial perfusion to the metastases, but after hepatic-artery ligation, portal-vein perfusion to the metastases was statistically significantly increased.**

**Hence, a compensatory haemodynamic mechanism exists which may account for the poor results of hepatic-artery ligation and perfusion alone.**

THE LIVER contains metastases in about one-third to one-half of fatal cases of colorectal malignance (Brown & Warren, 1938; Willis, 1948; Cedermark *et al.*, 1977). Goligher (1941) reviewed 893 cases coming to laparotomy and showed an incidence of 11·5%, whereas more recently Bengmark & Hafstrom (1969) found an incidence of hepatic metastases at laparotomy of 24·5% with a mean survival of 5·1 months. Other series have confirmed the bleak prognosis associated with colorectal liver secondaries (Pestana *et al.*, 1964; Jaffe *et al.*, 1968; Taylor, 1978) It has been estimated that as many as 50% of tumours will sooner or later give rise to liver metastases (Bengmark & Hafstrom, 1969).

The growth and development of liver metastases are dependent upon their blood supply. It is known that micrometastases reach the liver through the portal venous system by invasion of the mesenteric veins (Brown & Warren, 1938; Dukes, 1940; Fisher & Turnbull, 1955; Fisher & Fisher, 1959; Baserga *et al.*, 1960). While the developing metastases are small they continue to obtain their nutrition from the portal vein, but as they enlarge the blood supply is thought to be chiefly arterial (Ackerman, 1972). Breedis & Young (1954) studied blood flow into liver metastases in great detail, using injection of coloured dyes, chiefly in post-mortem specimens. Following injection into the hepatic artery the tumours were coloured, as was the adjacent normal liver, but the main bulk of the liver remained free. The conclusion was that the hepatic artery was the chief blood supply to liver metastases. Microscopic examination showed that the thin-walled portal vein (of all sizes) was occluded by the invading tumour cells so that pigment was prevented from reaching the metastases from portal vein injection.

These theories have led over the years to the development of hepatic artery ligation and various forms of cytotoxic perfusion techniques in the treatment of multiple colorectal liver metastases (Brennan *et al.*, 1963; Almersjo *et al.*, 1968, 1972; Watkins *et al.*, 1970; Murray-Lyon *et al.*, 1970; Ansfield *et al.*, 1971; Fortner *et al.*, 1973; Ansfield, 1975). Unfortunately, the results of these techniques in improving symptom-free survival have been particularly disappointing and unpredictable.

In this study we have measured blood flow into liver metastases, both at initial laparotomy and in the subsequent post-operative period, in patients undergoing surgery for colorectal cancer. Blood flow has been measured by the clearance of the inert radioactive gas xenon-133 administered into the liver. In this way, the relative perfusion characteristics of both normal liver and liver metastases before and after hepatic-artery ligation have been made. Since $^{133}$Xe is cleared at cellular level this method gives an indication of nutritive perfusion.

### MATERIALS AND METHODS

*Pre-operative measurements.*—These were obtained by direct parenchymal injection of $^{133}$Xe. Eight patients with macroscopically normal livers (controls), and 6 patients with extensive liver metastases were studied. The control subjects were patients undergoing cholecystectomy or vagotomy and pyloroplasty. The studies on patients with liver metastases were performed before palliative colorectal resections.

$^{133}$Xe dissolved in saline (0·1–0·15 ml = 100–15 $\mu$Ci) was directly injected into the liver substance with a fine needle. The needle was held in place for 30 s after injection to minimize escape of Xe via the needle tract. It was essential to ensure that no air bubbles entered the injectate since the Xe would preferentially enter the air bubbles rather than the venous return. The same type of needle was used for each injection.

A 1 in iodide detector with a multihole collimator was carefully placed over the area and held in a steady and constant position for 10 min. It was placed in such a way that it viewed the liver but not the lung, since this is the pathway of exit for the Xe. The detector was connected *via* a scaler ratemeter to a chart recorder with a chart speed of 5 cm/min. The time constant was 3 s and the count maintained at 6000 ct/s. Throughout the procedure the anaesthetic system was on an open circuit to allow the Xe to escape.

Recordings were obtained from the normal liver and from both normal liver and tumour in patients with metastatic deposits. Further recordings were obtained whilst the hepatic artery was occluded between fingers.

*Post-operative measurements.*—Dynamic blood-flow studies were performed on 17 patients who were included in a trial to assess different perfusion techniques for the treatment of multiple colorectal metastases noted at the time of initial laparotomy. In each case the primary tumour was resected and the patients were allocated to receive either no perfusion treatment, hepatic-artery ligation and distal cytotoxic perfusion (HAL + P), hepatic-artery ligation and distal cytotoxic perfusion with portal vein perfusion through the umbilical vein (HAL + P + UVP or umbilical-vein perfusion alone (UVP). The cytotoxic agent used was 5-fluorouracil and was given as a continuous perfusion into the umbilical vein, or in the case of the hepatic artery, into the distal vasculature after proximal ligation and division of the vessel.

Before removal of the catheters (on the tenth post-operative day) the patients were positioned supine under the gamma camera so that the anterior surface of the liver was in the field of view of the camera. Two mCi of $^{133}$Xe dissolved in saline was injected into the catheters and flushed through with 20 ml saline.

The rate of clearance of the $^{133}$Xe from the liver and metastases was recorded using an on-line computer system which collected the integrated counts over the field of view for 60 consecutive periods of 10 s. The resulting images were stored on magnetic disc. Each image, which consisted of an array of 64 × 64 elements, was reduced to an 8 × 8 array by summing over 64 adjacent points so that each new element represented a 3·5 × 3·5 cm area. Any element which represented areas within the field of view but outside the liver and which also contained radioactivity (*e.g.* the catheter and the lungs) were excluded from the subsequent analysis.

*Analysis of blood flow.*—Blood flow was expressed as liver perfusion in ml/min/100 g of liver tissue by multiplying the exponential rate constant of the fast component ($k$) by the partition coefficient ($\lambda$), *i.e.* blood flow $= k\lambda 100$.

The dynamic blood-flow images from the gamma camera were compared with $^{99m}Tc$ sulphur-colloid liver scans.

### RESULTS

### *Pre-operative blood flow*

*Normal liver.*—In 7 of the 8 patients studied a double-exponential clearance curve was obtained. The blood flow ranged from 36·6 to 123 mls/min/100 g (mean 73·4 ± 33·7 (Fig. 1). Temporary

FIG. 1.—Liver blood flow (in ml/min/100 g) obtained from both normal liver and liver metastases after direct parenchymal injection.

hepatic-artery occlusion with the fingers in 2 patients produced a fall in blood flow to 65% of the pre-occluded value.

*Metastatic nodules.*—Clearance after injection into metastatic nodules also gave the typical double-exponential curve. The mean values ranged between 7 and 78 ml/min/100 g (mean 41·5 ± 22·5). After hepatic artery occlusion with the fingers, perfusion through the nodules fell to a mean of 5% of the pre-occluded value (Fig. 2). On release of the occlusion, blood flow returned to pre-occlusion values.

### *Post-operative (dynamic studies)*

In each of the patients studied, a double-exponential clearance curve was apparent, both from the metastases and from the segments of normal liver. Analysis of the data showed 3 different types of image in the region of the metastases after $^{133}Xe$ administration.

(a) *The initial distribution image* showed where the isotope went to and reflected the distribution of blood vessels.

(b) *The perfusion image* was correlated by the computer and showed the areas of greater perfusion as the darker image.

(c) *The retention image* was also computer generated and showed regions in which the Xe clearance was prolonged for any reason (*i.e.* poorer perfusion through the tissue under study).

*Hepatic-artery ligation and distal cytotoxic perfusion (6 patients).*—The results in this group of patients were difficult to assess, since the hepatic artery was ligated and injection therefore made into the distal vasculature. In all patients the initial distribution was mainly to the liver metastases, and the hepatic-artery retention was high. The distribution image in such a patient is given in Fig. 3.

*Portal-vein perfusion alone (5 patients).*—In all patients the initial distribution of $^{133}Xe$ was to normal liver only (Fig. 4). In only 1 patient did a portal-vein perfusion image occur in a metastasis, and in this patient the retention image was prolonged, suggesting poor perfusion within the metastasis.

*Hepatic-artery ligation with cytotoxic perfusion and umbilical-vein perfusion (6 patients).*—The distribution images in this group of patients were particularly interesting. After injection into the hepatic-artery catheter, distribution was to the second deposits rather than normal liver (Fig. 5). This was associated in 4 patients with delayed retention images, suggesting poor perfusion.

FIG. 2.—Clearance curve after direct injection into a metastasis. Hepatic-artery occlusion with the fingers caused complete cessation of perfusion into the metastasis. On release of occlusion the blood flow returned to its pre-occluded rate. The graph reads from right to left.

However, in this group of patients, in whom the hepatic artery was ligated, injection of $^{133}$Xe into the portal-vein catheter produced distribution to both normal perfusion was even to both metastases and normal liver. In 4 patients, the retention image was poor, indicating increased blood flow.

### DISCUSSION

Two separate studies have been carried out to measure liver blood flow to different areas of the human liver, both with and without established liver secondaries. The method of measurement uses the clearance of $^{133}$Xe which is administered either by direct parenchymal injection into normal liver and adjacent liver metastases at laparotomy, or by intraportal and/or intrahepatic injections. Certain aspects of each technique require amplification.

Direct injection of $^{133}$Xe has previously been used to measure skeletal blood flow (Lassen, 1964) as well as liver blood flow (Gelin *et al.*, 1968). It has the advantage of simplicity and convenience, but suffers from the drawback of measurements only at laparotomy. However, it is a method which has proved both reliable at low flow levels and reproducible.

After injection, the Xe diffuses rapidly through the liver and very shortly after injection is in diffusion equilibrium with the tissues. However, blood leaving the capillaries and entering the venous return is also in equilibrium with the tissue,

(a)

(b)

FIG. 3.—(a) The pre-operative $^{99m}$Tc sulphur-colloid liver scan, showing liver metastases as filling defects. (b) After $^{133}$Xe injection into the hepatic artery, perfusion is chiefly to the metastases and surrounding normal liver.

hence injected Xe is in equilibrium with the liver and venous blood leaving the liver. The detector sees all the Xe injected into the liver. The blood flowing through the liver picks up the Xe and carries it away in the venous return, out of sight of the detector. Since the Xe is virtually completely expired when it reaches the lungs, there is little recirculation, hence the activity seen by the detector declines progressively. Gelin *et al.* (1968) have reported that measurements of blood flow obtained by direct injection of $^{133}$Xe into liver parenchyma gave a simple exponential curve. However, in this study a typical double-exponential

FIG. 4.—Distribution image after $^{133}$Xe injection into the portal-vein catheter. Note the perfusion to normal liver but minimal perfusion into the metastases.

curve in all but one patient has been obtained, suggesting that whatever the technique of $^{133}$Xe administration there is a double-exponential curve. Mackenzie *et al.* (1976) have shown that by screening out extra-hepatic radioactivity, the true intrahepatic clearance is mono-exponential.

Certain technical features also require emphasis. Firstly, small volumes of $^{133}$Xe are injected slowly, so that diffusion equilibrium is rapid and care is taken to ensure that no air bubbles are injected, since Xe will preferentially enter the air rather than the venous return. Secondly, the anaesthetic system must always be an open one, to allow the $^{133}$Xe to escape rather than be recirculated. Finally, since the lung is the pathway of exit for the Xe, the detector must be placed in such a way that it sees the liver but does not see the lung. It is conceivable that the anaesthetic agents will affect liver blood flow after direct injection and absolute values cannot be compared with the values in the conscious subject. Nevertheless, all the parameters will be altered similarly in all patients and the values obtained from each area of the liver are similarly affected.

The calculated blood flow will depend substantially on the partition coefficient

FIG. 5.—(a) The pre-operative $^{99m}Tc$ sulphur-colloid liver scan showing liver metastases as filling defects. (b) After $^{133}Xe$ injection into the hepatic artery, perfusion is again chiefly to the metastases. (c) Injection into the portal vein also produced a distribution image to the liver metastases.

of the tissue under study. In our measurements of partition coefficient by the technique of Veall & Mallett (1965) the values varied very little between normal and metastatic liver tissue.

Following direct $^{133}Xe$ injection, temporary occlusion of the hepatic artery reduced the blood flow in the normal liver to $\sim$ 65% of the unoccluded values, whereas flow to the large metastatic nodules was reduced to a mean of 5% of the pre-occluded value. On release of occlusion the blood flow returned to pre-occlusion values, which suggests both reliability and reproducibility of the technique. This confirms previous observations on the predominant hepatic arterial blood supply to liver metastases (Breedis and Young, 1954; Healey, 1965; Lien & Ackerman, 1970; Gelin *et al.*, 1968).

Several interesting observations were made in the dynamic blood-flow studies on the livers with widespread metastases. Firstly, in patients with portal-vein catheters alone, the blood flow was predominantly to normal liver, and perfusion within the secondary deposits was minimal. In the one patient with portal-vein perfusion, the $^{133}Xe$ retention image was prolonged suggesting a very sluggish blood flow. However, in patients in whom the main hepatic artery was ligated, portal-vein injection of $^{133}Xe$ produced distribution to secondary deposits in each of the 6 patients studied. There was clear perfusion to liver metastases in 5 patients, as well as to normal liver, and in 4 patients the retention image was poor, indicating increased blood flow.

Thus, after hepatic-artery ligation the portal-vein circulation appears to increase to the liver metastases almost as though there were a compensatory haemodynamic situation. Clearly the hepatic artery, when patent, contributes the majority of nutrition to liver metastases, with a minimal amount from the portal vein. But after occlusion of the main hepatic artery, the portal vein is able to increase its contribution to the metastases. This may help to explain the initial improvement after hepatic artery ligation reported in several series (Plengvanit *et al.*, 1967; Balasegaram, 1972) with diminution in size of liver metastases. However, a subsequent increase usually occurs with gradual clinical deterioration of the patient. Presumably this is due to the portal vein supplying nutrition to the metastases which leads to tumour growth.

Hence there may be good haemodynamic reasons for combining hepatic-artery ligation and perfusion with portal-vein perfusion. It may be of value to speculate on the possible compensatory factors which increase portal-vein perfusion to metastases after hepatic-artery ligation. It has been suggested that the thin-walled portal vein is compressed by the larger metastases and this occlusion explains the lack of portal flow to metastases (Lien & Ackerman, 1970). However, since the portal vein is able to increase its contribution after hepatic artery ligation, this is unlikely to be the entire explanation. The situation is comparable to the establishment of a "collateral" circulation. In other words, when the chief blood supply is compromised by deliberate occlusion of the hepatic artery, the portal vein is "stimulated" in some way to increase its contribution to the metastases. Lien & Ackerman (1970), studying implanted tumours in rats, showed that shunting from the hepatic artery to portal vein did occur within the tumour plexus, and that after hepatic-artery ligation the tumour-plexus circulation was filled *via* the portal vein.

Studies in normal liver have shown a widespread variation in blood flow between one area of the liver and another, and this variation alters from minute to minute (Sherriff *et al.*, 1977). It would appear reasonable, therefore, to suggest that the portal blood flow has the capacity to alter its rate substantially, presumably in response to various physiological situations. Hence, it may be that hepatic-artery occlusion is one of the many stimuli which produces an altered portal blood flow throughout the liver and liver metastases.

### REFERENCES

ACKERMAN, N. B. (1972) Alteration of intra-hepatic circulation due to increased tumour growth. *Proc. VIII Cong. Eur. Soc. Exp. Surg.* p. 182.

ALMERSJO, O., BENGMARK, S., ENGEVIK, L., HAFSTROM, L. LOUGHRIDGE, B. P. & NILSSON, L. A. V. (1968) Serum enzyme changes after hepatic dearterialization in man. *Ann. Surg.* **167**, 9.

ALMERSJO, O., BENGMARK, S., RUDENSTAM, C. M., HAFSTROM, L. & NILSSON, L. A. V. (1972) Evaluation of hepatic dearterialization in primary and secondary cancer of the liver. *Am. J. Surg.*, **124**, 5.

ANSFIELD, F. J. (1975) Further clinical studies with intra-hepatic arterial infusion with 5-fluorouracil. *Cancer*, **36** (Suppl), 2413.

ANSFIELD, F. J., RAMIREZ, G., SKIBBA, J. L. BRYAN, G. T., DAVIS, H. L. & WIRTANEN, G. W. (1971) Intra-hepatic arterial infusion with fluorouracil. *Cancer*, **28**, 1147.

BALASEGARAM, M. (1972) Complete hepatic dearterialization for primary carcinomas of the liver. Report of 24 patients. *Am. J. Surg.*, **124**, 340.

BASERGA, R., PUTONG, P. B., TYLER, S. & WARTMAN, W. B. (1960) The dose–response relationship between the number of embolic tumour cells and the incidence of blood borne metastases. *Br. J. Cancer*, **14**, 173.

BENGMARK, S. & HAFSTROM, L. (1969) The natural history of primary and secondary malignant tumours of the liver. 1. The prognosis for patients with hepatic metastases from colonic and rectal carcinoma by laparotomy. *Cancer*, **23**, 198.

BREEDIS, C. & YOUNG, G. (1954) Blood supply of neoplasms in the liver. *Am. J. Path.*, **30**, 969.

BRENNAN, M. J., TALLEY, R. W., DRAKE, E. H., VAITKEVICIUS, V. K., POZNANSKI, A. K. & BUSH, B. E. (1963) 5-Fluorouracil treatment of liver metastases by continuous hepatic artery infusion via Cournard catheter results and suitability for intensive post-surgical adjuvant chemotherapy. *Ann. Surg.*, **158**, 405.

BROWN, C. E. & WARREN, S. (1938) Visceral metastases from rectal carcinoma. *Surg. Gynecol. Obstet.*, **66**, 610.

CEDERMARK, B. J., SCHULTZ, S. S., BAKSHI, S. PARTHASARATHY, K. L., MITTELMAN, A. & EVANS, J. T. (1977) The value of liver scans in the follow-up study of patients with adenocarcinoma of the colon and rectum. *Surg. Gynecol. Obstet.*, **144**, 745.

DUKES, C. E. (1940) Cancer of the rectum; an analysis of 1000 cases. *J. Path. Bact.*, **50**, 527.

FISHER, B. & FISHER, E. R. (1959) Experimental studies of factors influencing hepatic metastases. III. Effect of surgical trauma with special reference to liver injury. *Ann. Surg.*, **150**, 731.

FISHER, E. R. & TURNBULL, R. B. (1955) The cytologic demonstration and significance of tumour cells in the mesenteric venous blood in patients with colorectal cancer. *Surg. Gynecol., Obstet.*, **100**, 102.

FORTNER, J. G., MULCARE, R. J., SOLIS, A., WATSON, R. C. & GOLBEY, R. B. (1973) Treatment of primary and secondary liver cancer by hepatic artery ligation and infusion chemotherapy. *Ann. Surg.*, **178**, 162.

GELIN, L., LEWIS, D. H. & NILSSON, L. (1968) Liver blood flow in man during abdominal surgery. I. Description of a method utilizing intrahepatic injections of radioactive xenon. Normal values and effect of temporary occlusion. *Acta Hepatogastroenterol.*, (*Stuttg.*) **15**, 13.

GOLIGHER, J. C. (1941) The operability of carcinoma of the rectum. *Br. Med. J.*, ii, 393.

HEALEY, J. E. (1965) Vascular patterns in human metastatic liver tumours *Surg. Gynecol. Obstet.*, **120**, 1187.

JAFFE, B. M., DONEGAN, W. L., WATSON, F. & SPRATT, JR. J. S. (1968) Factors influencing survival in patients with untreated hepatic metastases. *Surg. Gynecol. Obstet.*, **127**, 1.

LASSEN, N. A. (1964) Muscle blood flow in normal man and in patients with intermittent claudication evaluated by simulataneous $Xe^{133}$ and $Na^{24}$ clearance. *J. Clin. Invest.*, **43**, 1805.

LIEN, W. M. & ACKERMAN, N. B. (1970) The blood supply of experimental liver metastases. II. A microcirculatory study of the normal and tumour vessels of the liver with the use of perfused silicone rubber. *Surgery*, **68**, 334.

MACKENZIE, R. J., LEIBERMAN, D. P., MATHIE, R. T. RICE, G. C., HARPER, A. M. & BLUMGART, L. H. (1976) The liver blood flow measurement: the interpretation of $Xenon^{133}$ clearance curves. *Acta Chir. Scand.*, **142**, 519.

MURRAY-LYON, I. M., PARSONS, V. A., BLENDIS, L. M. & 4 others (1970) Treatment of secondary hepatic tumour by ligation of hepatic artery and infusion of cytotoxic drugs. *Lancet*, ii, 172.

PESTANA, C., REITEMEIER, R. J., MOERTEL, C. G & HAHN, R. G. (1964) The natural history of carcinoma of colon and rectum., *Am. J. Surg.*, **108**, 826.

PLENGVANIT, U., LIMWONGES, K., VIRANUVATTI, V. & KALAYASIRI, C. (1967) Treatment of primary carcinoma of the liver by hepatic artery ligation. Preliminary report of 40 cases. *Tijdschr. Gastroenterol.*, p. 490.

SHERRIFF, S. B., SMART, R. C. & TAYLOR, I. (1977) Clinical study of liver blood flow in man measured by $^{133}Xe$ clearance after portal vein injection. *Gut*, **18**, 1027.

TAYLOR, I. (1978) Cytotoxic perfusion for colorectal liver metases. *Br. J. Surg.*, **65**, 109.

VEALL, N. & MALLETT, B. L. (1965) The partition of trace amounts of xenon between human blood and brain tissue at 37°C. *Phys. Med. Biol.*, **10**, 375.

WATKINS, E., KHAZEL, A. M. & NAHRA, K. S. (1970) Surgical basis for arterial infusion chemotherapy of dissemmalect carcinoma of the liver. *Surg. Gynecol. Obstet.*, **130**, 581.

WILLIS, R. A. (1948) *The Pathology of Tumour*. London: Butterworth, p. 422.